# Optimization of Organotypic Cultures of Mouse Spleen for Staining and Functional Assays

**DOI:** 10.3389/fimmu.2020.00471

**Published:** 2020-03-24

**Authors:** Francesca Finetti, Nagaja Capitani, Noemi Manganaro, Vanessa Tatangelo, Francesca Libonati, Giulia Panattoni, Ivo Calaresu, Laura Ballerini, Cosima T. Baldari, Laura Patrussi

**Affiliations:** ^1^Department of Life Sciences, University of Siena, Siena, Italy; ^2^International School for Advanced Studies (SISSA/ISAS), Trieste, Italy

**Keywords:** organotypic culture, spleen, vibratome, precision-cut, white pulp, red pulp, lymphoma

## Abstract

By preserving cell viability and three-dimensional localization, organotypic culture stands out among the newest frontiers of cell culture. It has been successfully employed for the study of diseases among which neoplasias, where tumoral cells take advantage of the surrounding stroma to promote their own proliferation and survival. Organotypic culture acquires major importance in the context of the immune system, whose cells cross-talk in a complex and dynamic fashion to elicit productive responses. However, organotypic culture has been as yet poorly developed for and applied to primary and secondary lymphoid organs. Here we describe in detail the development of a protocol suitable for the efficient cutting of mouse spleen, which overcomes technical difficulties related to the peculiar organ texture, and for optimized organotypic culture of spleen slices. Moreover, we used microscopy, immunofluorescence, flow cytometry, and qRT-PCR to demonstrate that the majority of cells residing in spleen slices remain alive and maintain their original location in the organ architecture for several days after cutting. The development of this protocol represents a significant technical improvement in the study of the lymphoid microenvironment in both physiological and pathological conditions involving the immune system.

## Introduction

Organotypic culture has emerged as a powerful technique which allows the analysis of tissue behavior in a variety of conditions. Initially developed as an alternative to classical 2-D *in vitro* culture of neurons, slices obtained by sectioning the brain region of interest with tissue choppers allowed to maintain neurons alive outside of the body and were found to be suitable for electrophysiological studies (1). Preparation and *in vitro* growth of these slices were progressively optimized to be maintained in culture for several days thanks to the introduction of modern tissue choppers such as vibrating microtomes, that produce thinner and less damaged slices, and tissue support systems, such as agarose, to preserve the 3-D organization of the tissue (1, 2).

Organotypic culture has been extended to several other organs of the neuroendocrine system (1) and, more recently, to tumor-derived tissues (3). Of note, only one report describes the application of this technique to lymphoid tissues of human origin (4), notwithstanding the wealth of information generated over the last decade on the complex interactions that occur among immune, stromal, and cancer cells (5, 6). Cancer immunotherapies, now applied to a variety of cancers, often result in heterogeneous responses, to which the specific features of the individual tumor microenvironment may contribute (7). Hence, the optimization of organotypic culture of lymphoid organs is critically important to understand the immune cell microenvironment in a variety of tumors.

Efficient preparation of spleen slices faces the challenge posed by the complex structure and texture of this lymphoid organ. The spleen is indeed organized as a “tree” of branching arterial vessels, in which the smaller arterioles end in a venous sinusoidal system. The organ is surrounded by a fibrous capsule of connective tissue, from which the connective trabeculae protrude into the splenic tissue to support vessels (8). Due to this peculiar organ texture, preparation of spleen slices with a chopper is precluded. Spleen is crushed by the blade and slices are not useful for further analyses (unpublished observations).

We developed a new protocol that allowed us to efficiently cut mouse spleens in intact slices and to maintain these alive and responsive for at least 48 h, making them suitable for functional assays. The protocol, that is a modification of protocols developed to obtain precision-cut slices of mouse brain, liver and lung (9–11), is based on the sequential following steps: (1) spleen inclusion into agarose blocks; (2) precision-cut using a vibrating microtome; and (3) 48-h culture of spleen slices. The protocol developed for the preparation of organotypic cultures of mouse spleens has turned out to be a valuable tool to (i) prepare spleen slices with a sufficient degree of tissue integrity; and (ii) maintain this complex tissue in culture for days, in order to be used for functional assays.

## Materials and Equipment

### Spleen Harvesting

Scissors, micro-dissecting forceps, 2-ml polypropylene microtubes (Sarstedt), ice box, ice. Culture medium: high glucose Dulbecco Modified Eagle's Medium (DMEM) (Sigma-Aldrich) with 2 U/ml penicillin G (Sigma-Aldrich) and 7.5% bovine calf serum (BCS, Hyclone).

### Precision-Cut of Mouse Spleen

Scissors, curved micro-dissecting forceps, scalpel, small spatula, plastic film, tweezers, agarose, thermometer, microwave, 50 ml beaker, milliQ water, phosphate-buffered saline (PBS), 3.5 ml transfer-pipette (Sarstedt), Compresstome® VF-300-0Z Vibrating Microtome with Specimen tube and Syringe chilling block (Precisionary instruments, Greenville, NC, USA), diagnostic microscope slides (Menzel Glaser- Thermo Scientific), pipettes, pipette tips.

### Culture of Spleen Slices

Laminar airflow chamber, sterile 48-well plates with flat bottom (Sarstedt), cell culture incubator with 5% CO_2_. Culture medium: high glucose Dulbecco Modified Eagle's Medium (DMEM) (Sigma-Aldrich) with 2 U/ml penicillin G (Sigma-Aldrich) and 7.5% BCS (Hyclone).

## Methods

### Animals

C57BL/6J mice were housed in a pathogen-free and climate-controlled (20 ± 2°C, relative humidity 55 ± 10%) animal facility at the University of Siena. Mice were provided with water and pelleted diet *ad libitum*. All cages are provided with environmental enrichment in the form of nesting material and mouse houses. Procedures and experimentation were carried out in accordance with the 2010/63/EU Directive and approved by the Italian Ministry of Health. Animals were euthanized and spleens were harvested, immediately transferred to ice-cold culture medium (see “Materials” section) and stored on ice.

### Slice Stimulation, RNA Purification, and RT-PCR

RNA extractions were carried out on samples composed of 1, 3, or 5 spleen slices. Samples were homogenized in 1.5 ml microtubes using polypropylene double-ended pestle (Sigma-Aldrich) in 350 μl RLT lysis buffer of the RNeasy Mini Kit (Qiagen) until completely homogenized. RNA was then extracted and retrotranscribed as described (12). RNA amount and quality were assessed using QIAxpert System (Qiagen). Real-time PCR was performed in triplicate on 96-well optical PCR plates (Sarstedt AG, Nümbrecht, Germany) using SSo FastTM EvaGreenR SuperMix (Biorad Laboratories, Hercules, CA) and a CFX96 Real-Time system (Bio-Rad Laboratories, Waltham, MA). Results were processed and analyzed as described (12). Transcript levels were normalized to GAPDH. Spleen slices (3 slices per sample) either freshly cut or cultured for 48 h at 37°C in culture medium were stimulated with A23187 (Merck, cat. C7522, 500 ng/ml) and phorbol 12-myristate 13-acetate (PMA, Merck, cat. 524400, 100 ng/ml) in culture medium for 6 h at 37°C, homogenized in 350 μl RLT lysis buffer and RNA was extracted as described above. Primers used for amplification were: mouse CCL19 Forward 5′-3′, CAA GAA CAA AGG CAA CAG C; mouse CCL19 Reverse 5′-3′, CGG CTT TAT TGG AAG CTC TG; mouse CXCL13 Forward 5′-3′, CAT CAT GAG GTG GTG CAA AG; mouse CXCL13 Reverse 5′-3′, GGG TCA CAG TGC CAA AGG AAT; mouse GAPDH Forward 5′-3′, AAC GAC CCC TTC ATT GAC; mouse GAPDH Reverse 5′-3′, TCC ACG ACA TAC TCA GCA C; mouse IL-2 Forward 5′-3′, CCC TTG CTA ATC ACT CCT CA; mouse IL-2 Reverse 5′-3′, GAA GTG GAG CTT GAA GTG GG; mouse IL-10 Forward 5′-3′, CCG GAC AGC ACA CTT CAC AG; mouse IL-10 Reverse 5′-3′, TCC ACC ATT TCC CAG ACA AC; mouse IFN-γ Forward 5′-3′, ACT GGC AAA AGG ATG GTG AC; mouse IFN-γ Reverse 5′-3′, AAA CTT GGC AAT CTC ATG AAT G.

### Optical and Immunofluorescence Microscopy

Spleen slices were carefully placed on diagnostic microscope slides (Menzel Glaser-Thermo Scientific), left either unlabeled or stained for 8 min with Trypan blue solution 0.4% (Sigma-Aldrich) diluted 1:2 in PBS, washed with PBS until complete removal of the exceeding dye, covered with 24 × 60 mm coverslips (VWR) and observed with SZX12 stereo light microscope (Olympus) and DMRB microscope (Leica microsystems) equipped with Zeiss AxioCam MRc5 digital camera. Images were processed using the AxioVision Rel. 4.6.3. software.

Immunofluorescence microscopy was performed following a modification of the protocol previously described (13). Briefly, spleen slices were transferred with a small spatula to 10-well diagnostic microscope slides (Thermo Scientific), one slice/well, and incubated for 30 min at RT with 30 μl fixation buffer (4% paraformaldehyde in PBS) in the dark, washed with PBS and incubated for 30 min at RT with 30 μl permeabilization solution (PBS 0.1% BSA plus 0.01% Triton X-100). Slices were then stained with 30 μl/well of either unconjugated primary Ab or fluorescently labeled Ab in Hanks' salts at RT in the dark for 2 h, washed with PBS and incubated with 30 μl/well of fluorochrome-conjugated secondary antibodies at RT in the dark for 2 h. Slides were washed with Hanks' salts; mounting medium (PBS 90% glycerol) was added and slides covered with 24 × 60 mm coverslips (VWR) and sealed with conventional nail polish. Images were acquired on Zeiss LSM700 confocal microscope using 63 ×, 40 × and 10 × objectives. Primary antibodies: FITC Rat anti-mouse CD19 (BD Pharmingen, cat. 553758) 1:30 in Hanks' salts; Alexa Fluor 488 anti-mouse CD3ε (eBiosciences, cat. 53-0031-82) 1:30 in Hanks' salts; Rat anti-mouse ER-TR7 (ABD Serotec, cat. MCA2402) 1:50 in Hanks' salts; mouse monoclonal anti-Follicular DC Marker (Santa Cruz, cat. sc-58529, Ki-M9R clone) 1:50 in Hanks' salts. Secondary antibodies: DyLight® goat anti-rat 488 (Bethyl, cat. A110-105-D2) and 550 (Bethyl, cat. A110-105-D3) 1:100 in Hanks' salts; Alexa Fluor goat anti-mouse 488 (Thermo-Fisher scientific, cat. A-11001) 1:100 in Hanks' salts; isotype control: FITC Rat IgG2a (BD Pharmingen, cat. 553924).

### Flow Cytometry, Chemotaxis Assays, and Trypan Blue Exclusion

Spleen slices were disgregated using 70-μm Cell strainer filter (BD Falcon™) and 1 ml syringe (BioSigma). Cell death was measured by flow cytometry on slice-derived splenocytes by quantifying the % of either Annexin V^+^/Propidium Iodide (PI)^−^ or PI^+^ cells as described (12). Briefly, 2 × 10^5^ cells were resuspended in 200 μl PBS and stained with Annexin V FITC (eBiosciences) for 15 min at RT. When required, PI was added to the samples at the final concentration of 10 ng/ml immediately before the flow cytometric analysis using Guava Easy Cyte (Millipore, Billerica, MA) cytometer. Alternatively, cells were stained with Annexin V PE (eBiosciences), fixed (fixation buffer, 4% paraformaldehyde in PBS) and permeabilized (permeabilization solution, PBS 0.1% BSA plus 0.01% Triton X-100), and T lymphocytes, B lymphocytes, follicular dendritic cells (FDC) or reticular fibroblasts were stained with anti-mouse CD3ε (eBiosciences) 1:30 in PBS, Rat anti-mouse CD19 (BD Pharmingen) 1:30 in PBS, anti-Follicular DC Marker 1:50 in PBS, and Rat anti-mouse ER-TR7, respectively. Surface CXCR4 and CCR7 were stained with either Rabbit anti-CXCR4 antibody (Abcam, cat. AB124824), 1:50 in PBS, or Rabbit monoclonal anti-CCR7 antibody (Novus Biologicals, cat. NB110-55680, Y59 clone), 1:50 in PBS, and Alexa Fluor goat anti-rabbit 488 secondary antibodies (Thermo-Fisher scientific, cat. A-11002) 1:400 in PBS, in combination with PE Rat anti-mouse CD3ε (eBiosciences, cat. 145-2C11, 2C11 clone) 1:30 in PBS or PE Mouse anti-mouse CD22.2 (BD Pharmingen, cat. 553384, Cy34.1 clone) 1:30 in PBS, and analyzed by flow cytometry. Chemotaxis assays were modified from the protocol reported in (14). Briefly, spleen slices were placed on the upper well of Boyden chamber, and allowed to position over the porous membrane of the insert. CXCL12 (Merck, cat. SRP4388, 100 ng/ml) or MIP-3β (Merck, cat. SRP4495, 100 ng/ml) were diluted in culture medium and placed in the lower well of the chamber. Cells were allowed to migrate for 3 h at 37°C, then the migrated cells were recovered from the lower chamber and stained with PE Rat anti-mouse CD3ε (eBiosciences) 1:30 in PBS and FITC Rat anti-mouse CD19 (BD Pharmingen) 1:30 in PBS, and analyzed by flow cytometry. Slice-derived splenocytes were stained for 8 min with Trypan blue solution 0.4% (Sigma-Aldrich) diluted 1:2 in PBS and Trypan blue^+^ cells were counted using an optical microscope. The percentage of dead cells was assessed by calculating the percentage of Trypan blue^+^ cells over the total cell count.

### Statistical Analyses

Mean values, standard deviations and Student's *t*-test were calculated using GraphPad (Prism 7). A level of p<0.05 was considered statistically significant.

### Stepwise Procedure for Preparation and *in vitro* Culture of Spleen Slice

#### Spleen Preparation for Sectioning

- Lay down spleen on clean plastic film using tweezers or curved micro-dissecting forceps;
- Carefully remove fat, fur and debris using scissors and/or scalpel;
- Immediately transfer spleen into ice-cold culture medium until next step.

#### Spleen Inclusion into the Agarose Block

**Figure 1:**
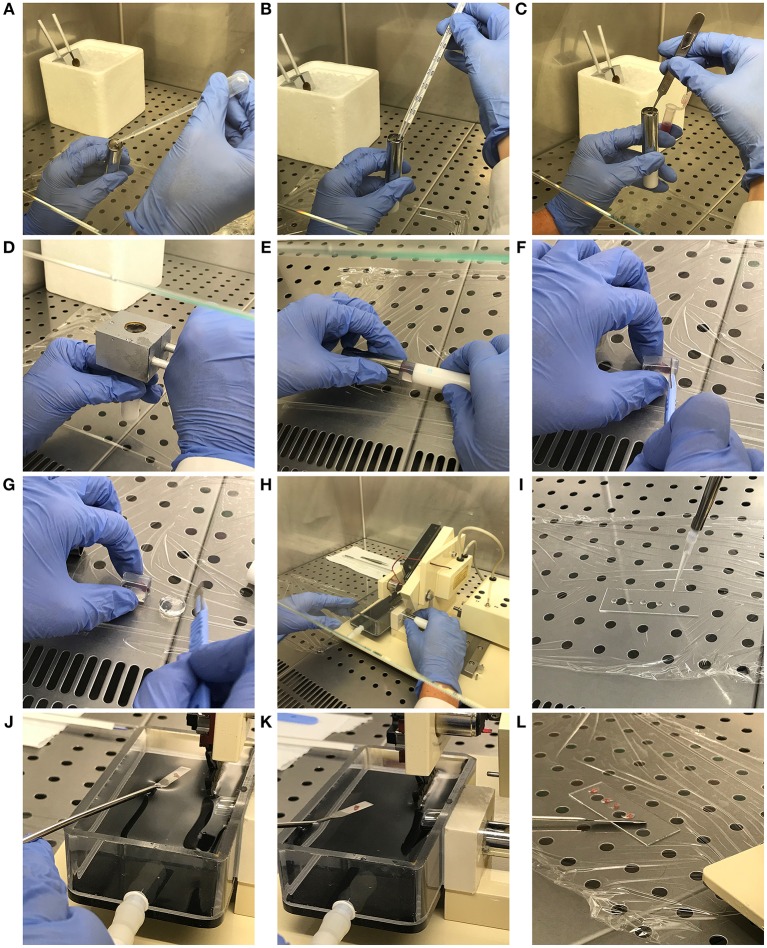
Mouse spleen inclusion in agarose and precision-cut: step-by-step description of the protocol. **(A)** Syringe chilling block pre-cooling and specimen tube filling. **(B)** Agarose temperature checking. **(C)** Spleen inclusion into the agarose block. **(D)** Chilling of the spleen-containing agarose block. **(E)** Extraction of the agarose block containing the embedded sample. **(F,G)** Exceeding agarose removal from the agarose block. **(H)** Specimen tube insertion into the sample housing. **(I)** Preparation of the microscope slide with PBS drops. **(J,K)** Spleen slice preparation and recovery from the buffer tank with a spatula. **(L)** Spleen slice transfer in the PBS drops of the microscope slide.

- Set up Compresstome® VF-300-0Z Vibrating Microtome (Precisionary Instruments, Greenville, NC, USA) following the manufacturers' instructions;
- Pre-chill syringe chilling block (Precisionary Instruments, Greenville, NC, USA) in ice for at least 10 min;
- Wash diagnostic microscope slides (Thermo Scientific) with MilliQ, then wash with absolute ethanol and allow them to air-dry;
- In a 50 ml beaker prepare 3% agarose solution in MilliQ, melt it by microwave and allow it to cool down to 45°C at room temperature, repeatedly checking the temperature with thermometer and shaking the solution to avoid agarose clumps;
- Open the provided specimen tube (Precisionary Instruments, # VF-SPS-VM-12.5) and fill it with 3–4 ml of 45°C agarose solution (Figure 1A). Check the temperature with thermometer until it reaches 38°C (Figure 1B).
- Carefully pick up the spleen from ice-cold culture medium using tweezers and rapidly insert it vertically into the agarose-containing specimen tube. Pay attention to maintain the spleen in a vertical position during this step (Figure 1C).
- Immediately place the pre-chilled syringe chilling block (see step 3.6.2.2) over the specimen tube and leave immobile for at least 2 min (Figure 1D). This process accelerates agarose solidification and chills the whole sample.

#### Preparation of Spleen Slices

- Extract the agarose block containing the embedded sample from the specimen tube and place it on clean plastic film (Figure 1E). Proceed to remove the portions of agarose which do not contain organ portions with the scalpel, carefully placing the blade perpendicular to the agarose block (Figures 1F,G). Place the carved agarose block back in the specimen tube. This step allows the specimen tube containing agarose-embedded full-length spleen to fit with the Motor Box plunger of the Compresstome.
- Insert the specimen tube in the housing and fill the buffer tray with sterile PBS (Figure 1H).
- In order to obtain non-damaged spleen slices, set the cut speed to the minimum and the blade oscillation to the maximum in the instrument control box. Importantly, set the slice thickness to ~ 230 μm. This is absolutely required to obtain good quality spleen slices. Lower thickness results in profound damage of the spleen structure, while higher thickness does not allow microscope observation and analyses.
- Proceed to spleen slice preparation following the technical instructions provided by the manufacturer.
- While proceeding with tissue cut, place 4–5 drops of PBS in each washed microscope slide with a 200 μl pipette (Figure 1I).
- Recover spleen slices using a spatula (Figures 1J,K) and transfer them in the PBS drops (Figure 1L), carefully removing any trace of agarose.

#### Culture of Spleen Slices

- Transfer slices from the microscope slide to a sterile 48-well plate with flat bottom (Sarstedt) containing 100 μl culture medium in a laminar airflow chamber under sterile conditions (one or more slides per well).
- Maintain in cell culture incubator at 37°C and with 5% CO_2_.
- Check the volume of culture medium every day and be careful not to exceed 100 μl to avoid oxygen deprivation. In case of evaporation, add just the culture medium amount required to cover the slice.

### Critical Parameters and Trouble Shooting

The efficiency of the whole process primarily depends on step 3.6.2., which describes the building of the agarose block containing the spleen (Figures 1A–D). The temperature of the agarose solution must not exceed 40°C and should ideally be maintained around 38°C to avoid organ damage. Moreover, this temperature allows optimal insertion of the spleen into the agarose, which then quickly polymerizes thereby maintaining the organ in the exact position and vertical orientation where it was placed. This is particularly important since this bean-like organ tends to rotate and lie down over the specimen tube when the agarose solution is too fluid, thereby precluding well-oriented spleen cut.

Non-intact slides, such as slides with breaks in the spleen capsule, must not be used for further analyses since they do not maintain the original organ architecture and they rapidly crinkle. In order to preserve slice integrity, fat and other debris must be removed from the spleen before cut, and air bubble formation in the agarose during the inclusion step must be avoided. Small bubbles are nevertheless quite common since tweezers, sometimes immersed for a few seconds into agarose to maintain the spleen vertical, generate bubbles when extracted from the solidifying agarose.

Although some slices are still included in the polymerized agarose when transferred to the PBS-containing buffer tray, the majority detach from the agarose disk during cut and float in the PBS, making it extremely hard to retrieve them without inflicting severe damages to the organ structure. We adjusted this step of the protocol using a small spatula to get close to and capture even the smaller PBS-floating slices.

## Results

### Spleen Slices Cultured for 48 h *in vitro* Preserve Organ Integrity

**Figure 2:**
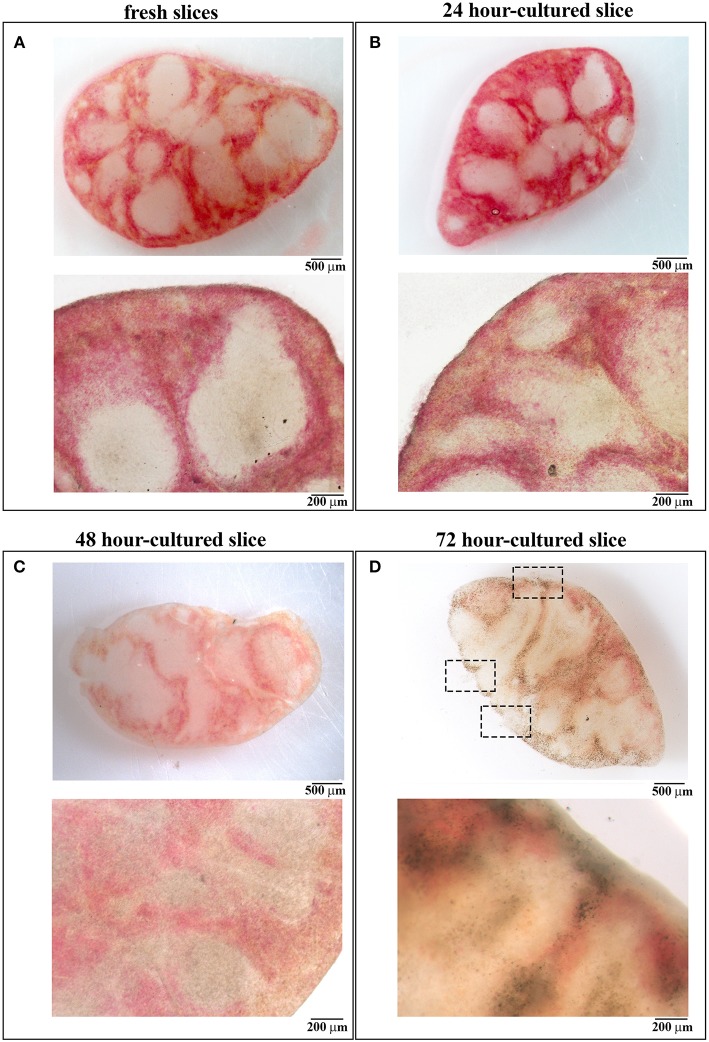
48 h-cultured spleen slices maintain unaltered their architecture. **(A–D)** Images obtained by stereomicroscopy (upper panels) and optical microscopy (lower panels) of spleen slices either immediately after cutting **(A)** or after *in vitro* culture for 24 **(B)**, 48 **(C)**, and 72 **(D)** hours. Representative images are shown (*n* = 3). Size bars are indicated.

Slices obtained applying the protocol described in the “Methods” section were transferred to diagnostic microscope slides and either left unstained or stained with Trypan blue. Optical microscopy was used to evaluate organ architecture. As shown in Figure 2, fresh slices were intact and the splenic structure was unaffected by the cut, with red pulp spacing out wide white pulp areas (Figure 2A).

Slices were cultured for 24, 48, 72, and 96 h in 48-well plates at 37°C with culture medium, one slice per well. To evaluate whether the *in vitro* culturing procedure affects organ architecture, cultured slices were transferred to diagnostic microscope slides and subjected to optical microscopy. As shown in Figure 2, slices cultured for 24 and 48 h were intact and both the splenic structure and the red-white pulp ratio remained almost unchanged when compared to fresh tissues (Figures 2B,C). Beginning from 72 h we observed a high degree of disruption of the slice architecture with wide necrotic zones affecting both the red and the white pulp (Figure 2D) and small ruptures lacerating both the organ texture and the surrounding capsule (see dashed rectangles in Figure 2D), possibly due to extreme slice fragility. We were unable to perform optical microscopy of 96 h-cultured slices, which were extremely damaged during their transfer to the microscope slides (data not shown). Our data suggest that 48 h is the longest time point of *in vitro* culture which maintains unaffected the architecture of spleen slices.

### 48 H-Culture Does Not Significantly Affect Cell Viability of Spleen Slice Cells

**Figure 3:**
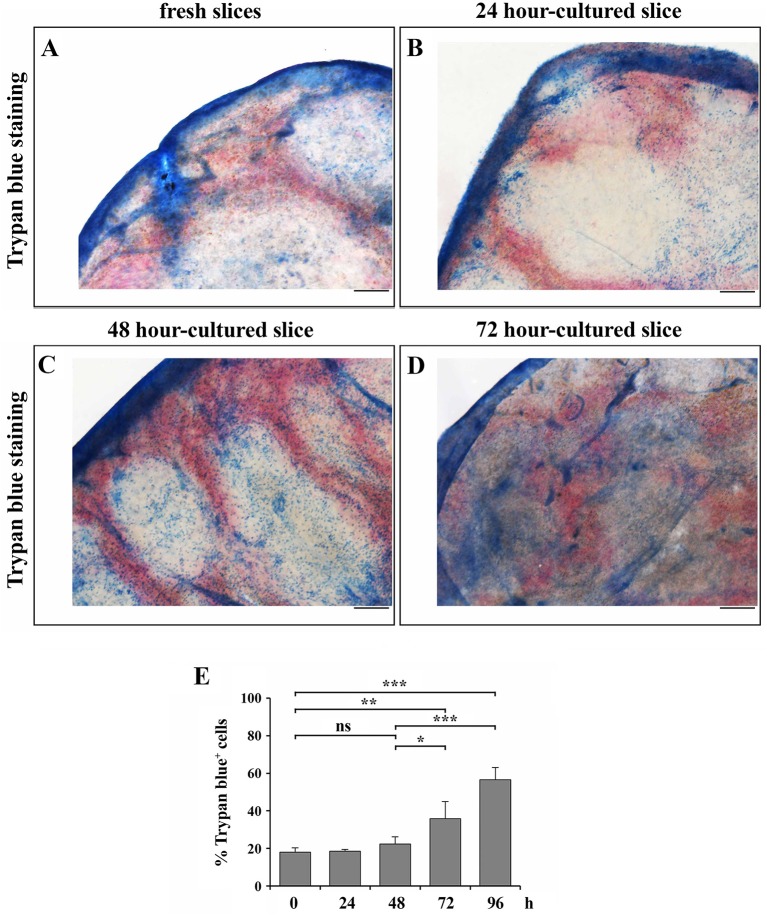
48 h-culture does not significantly increase cell death of spleen slices. **(A–D)** Optical microscopy pictures of spleen slices either immediately after cutting **(A)** or cultured *in vitro* for 24 **(B)**, 48 **(C)**, and 72 **(D)** h and stained with Trypan blue. Representative images are shown (*n* = 3). Size bar, 200 μm. **(E)** Trypan blue exclusion assay performed on spleen slices disgregated either immediately after cutting (0) or after *in vitro* culturing for 24, 48, 72, and 96 h, stained with Trypan blue and counted by optical microscopy. The data are presented as the percentage of Trypan blue^+^ cells over total cells (*n* = 3). Mean ± SD. Student's *t*-test; ***p ≤ 0.001; **p ≤ 0.01; *p ≤ 0.05.

In an attempt to understand whether 48 h-cultured slices also maintain a reasonable degree of cell viability, we analyzed cell death in spleen slices either freshly prepared or cultured for 24, 48, 72, or 96 h at 37°C. Slices were transferred to diagnostic microscope slides, either left unstained, or stained with Trypan blue and then analyzed by optical microscopy. Trypan blue staining of spleen slices showed substantial non-specific staining of the trabecular outer capsule, which occurred independently of the culture time (Figures 3A–D). Trypan blue-positive cells could be observed in both red and white pulp of fresh slices (Figure 3A) and in samples cultured for 24 and 48 h, where staining was not significantly different from staining performed on fresh samples (Figures 3A–C). In contrast, spleen slices cultured for 72 h showed stronger Trypan blue positivity (Figure 3D), which suggests a high degree of cell death. As reported above, we were unable to perform Trypan blue staining in 96 h-cultured slices, since they broke down during their transfer to the microscope slides.

**Figure 4:**
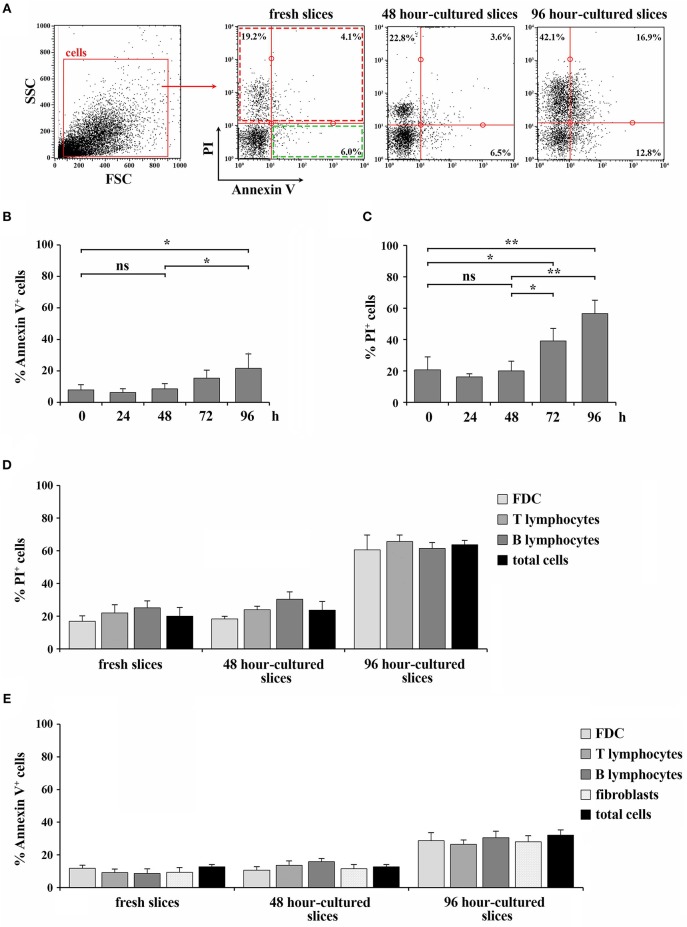
48 h-culture does not selectively affect the viability of cell types within spleen slices. **(A–C)** Flow cytometric analysis of the percentage of Annexin V^+^ cells **(B)** or PI^+^ cells **(C)** in spleen slices disgregated either immediately after cutting (fresh slices, 0) or after *in vitro* culturing for 24, 48, 72, and 96 h, and stained with Annexin V FITC and with 10 ng/ml PI. The data are presented as the percentage of either Annexin V^+^ or PI^+^ cells over total cells (*n* = 3). Representative flow cytometric plots are shown in **(A)**. **(D)** Flow cytometric analysis of spleen slices either immediately after cutting (fresh slices) or cultured *in vitro* for 48 h (48 h-cultured), disgregated, labeled for CD3, CD19, or FDC marker and with 10 ng/ml PI. **(E)** Flow cytometric analysis of spleen slices either immediately after cutting (fresh slices) or cultured *in vitro* for 48 h (48 h-cultured), disgregated, labeled for CD3, CD19, FDC, or ER-TR7 and Annexin V PE. The data are presented as the percentage of either PI^+^ **(D)** or Annexin V^+^ **(E)** cells over CD3^+^, CD19^+^, FDC^+^, or ER-TR7^+^ cells (*n* = 4). Mean ± SD. Student's *t-*test; **p ≤ 0.01; *p ≤ 0.05.

To quantify the extent of cell death, freshly prepared or cultured spleen slices were disgregated and Trypan blue exclusion assays were performed. In line with the results obtained by optical microscopy, the percentages of Trypan blue^+^ cells in 24- and 48 h-cultured slices were comparable to fresh tissues, while longer culture times elicited significantly higher positivity, with a very high percentage of Trypan blue^+^ cells in 96 h-cultured slices, indicating a high degree of cell death in slices cultured for over 48 h (Figure 3E). The extent of cell death was also quantified by flow cytometry in spleen slices either freshly prepared or cultured for 24, 48, 72, or 96 h at 37°C, disgregated and stained with Annexin V and PI. As shown in Figure 4, slices cultured for 24 and 48 h showed percentages of PI^+^ dead cells, as well as of Annexin V^+^/PI^−^ early apoptotic cells, comparable to freshly cut slices (Figures 4A–C). By contrast, the percentage of Annexin V^+^/PI^−^ early apoptotic cells and, to a higher extent, of PI^+^ dead cells increased in slices cultured at 37°C for longer times (Figures 4A–C). These results indicate the beginning of the deterioration process in slices subjected to prolonged *in vitro* culture. To investigate whether the viability of the different cell types present in the slices was differentially affected during slice culture, we carried out a flow cytometric analysis of spleen slices either freshly prepared or cultured for 48 or 96 h at 37°C, disgregated and stained with PI in combination with antibodies against CD3, CD19, and FDC, which specifically stain the spleen-resident T cells, B cells and FDCs, respectively. As shown in Figure 4D, the deterioration process equally affects all cell types analyzed. Staining of spleen-resident reticular fibroblasts was carried out using an antibody against the specific cytoplasmic marker ER-TR7. This requires plasma-membrane permeabilization, which prevents labeling of dead cells with PI. To overcome this limitation the relative levels of death in spleen-resident cell populations was also evaluated by staining slice-derived cells with the surface apoptotic marker Annexin V in combination with antibodies against CD3, CD19, FDC, and ER-TR7. As shown in Figure 4E, the percentage of Annexin V^+^ cells was similar among cell types and did not significantly change compared to total cells. Collectively, our data demonstrate that while 48 h-cultured slices maintain the correct tissue organization and do not display an enhanced degree of cell death compared to freshly cut slices, 72 h-cultured slices appear significantly damaged, indicating that spleen slices can be maintained in culture for no more than 48 h, at least in the culture conditions used.

### Spleen Slices Cultured for 48 h *in vitro* Maintain the Tissue Localization of Cell Populations

**Figure 5:**
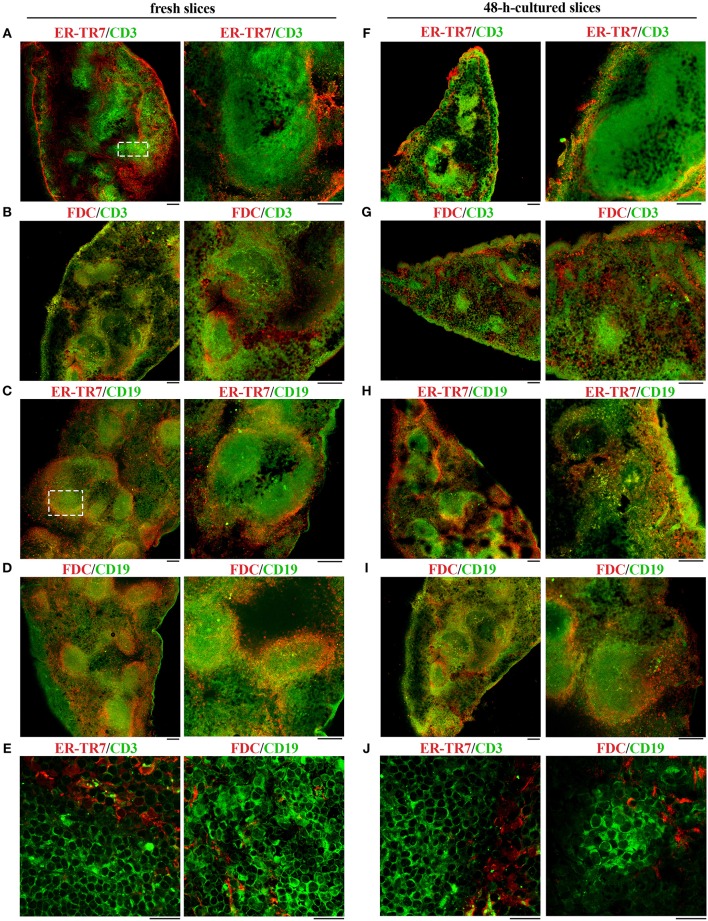
48 h-culture does not alter the distribution of cell populations in spleen slices. Immunofluorescence analysis of spleen slices either immediately after cutting (fresh slices, **A**–**E**) or cultured *in vitro* for 48 h (48-h-cultured, **F**–**J**), permeabilized and labeled for ER-TR7 (red)/CD3 (green) **(A,E,F,J)**, ER-TR7 (red)/CD19 (green) **(C,H)**, FDC marker (red)/CD3 (green), **(B,G)** or FDC marker (red)/CD19 (green) **(D,E,I,J)**. The pictures refer to non-contiguous spleen slices. Median optical sections are shown. Size bar, 100 μm **(A–D,F–I)**, and 20 μm **(E,J)**.

The lymphoid tissue that constitutes the white pulp is organized around the arterial vessels in T- and B-cell compartments, whose maintenance is controlled by specific chemokines that attract T and B cells to their respective localization (8, 15). To evaluate the localization of immune cells and to assess the extent of organ texture degeneration in spleen slices subjected to prolonged *in vitro* culturing, cell distribution was assessed by immunofluorescence in spleen slices either immediately after cutting (fresh slices) or cultured for 48 h at 37°C in culture medium (Figures 5A–J and Supplementary Figure 1). In fresh slices immune cells were mainly distributed in lymphoid compartments, with T cells localized in T cell areas (Figure 5A, dashed rectangle), B cells mainly confined to germinal centers (Figure 5C, dashed rectangle), with a framework of ER-TR7-secreting reticular fibroblasts and FDC surrounding the marginal zones (Figures 5A–E). This structure was maintained almost unchanged in slices cultured for 48 h (Figures 5F–J), although we observed a partial decrease in the size (panel G) and/or cellularity (panels G-I) of white pulp areas, which might be accounted for by a partial loss of non-adherent lymphocytes in the culture medium. These results, together with the fact that we were unable to perform the immunofluorescence analysis of 96 h-cultured slices due to their high degree of degeneration, suggest that 48 h-cultured spleen slices are suitable for functional assays.

### RNA Extracted From 48 H-Cultured Spleen Slices Is Suitable for qRT-PCR Analysis

**Figure 6:**
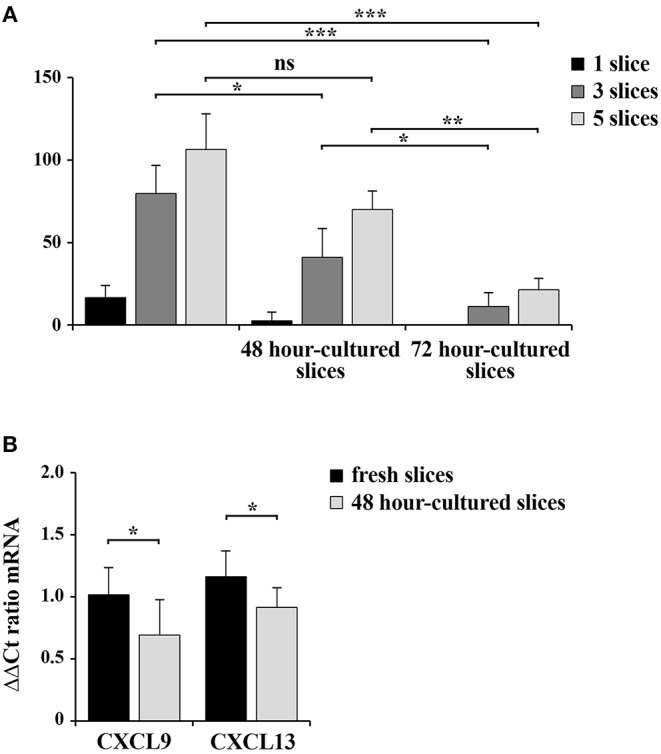
48 h-cultured spleen slices show a slight decrease in RNA yield and quality. **(A)** RNA concentration in samples from 1, 3, or 5 spleen slices homogenized in RNA extraction buffer either immediately after cutting or after *in vitro* culturing for 48 and 72 h. The measurement of RNA concentration was performed using QIAxpert System (Qiagen). **(B)** Quantitative RT-PCR analysis of CCL19 and CXCL13 mRNA in samples from 5 spleen slices homogenized either immediately after cutting or after *in vitro* culturing for 48 h. The relative gene transcript abundance was determined on triplicate samples using the ddCt method. *Gapdh* was used as housekeeping gene. Mean ± SD. Student's *t*-test; ***p ≤ 0.001; **p ≤ 0.01; *p ≤ 0.05.

RNA was extracted from homogenates obtained from 1, 3, and 5 spleen slices either immediately after cutting (0 h) or maintained in culture for 48 and 96 h. RNA was quantified and its quality checked using QIAxpert System (Qiagen). As shown in Figure 6A and in Table 1, the amount of RNA was very low when extracted from 1 slice immediately after cut. Conversely, both the yield and the quality of RNA extracted from 3 and 5 slices were sufficient to allow for retrotranscription (Figure 6A and Table 1). Similar results were obtained when we analyzed samples cultured for 48 h, where we observed a limited decrease in both yield and quality of RNA (Figure 6A and Table 1). As expected, RNA recovery in 96 h-cultured slices was very low (Figure 6A and Table 1). Based on these results, the quality of the RNA was assessed by qRT-PCR analysis on RNA obtained from 5 slices either fresh or cultured for 48 h. In order to define the sensitivity of the assay, the transcripts encoding for the chemokines CCL19 (CC–chemokine ligand) and CXCL13 (CXC–chemokine ligand) were selected, due to their very low expression levels in lymphoid tissues under physiologic conditions (16). GAPDH was used as the housekeeping gene. The transcripts for both CCL19 and CXCL13 were easily detectable in fresh slices and were still clearly detectable in slices cultured for 48 h, although slightly decreased (Figure 6B) despite the lower RNA yield of these cultured samples (Figure 6A). Collectively, our data highlight the advantages of the new method described here to obtain live spleen sections which preserve both structure and cell viability and that can be used for functional assays.

**Table 1:** Yield and quality of RNA isolated from spleen slices (1, 3, or 5 slices/sample) immediately after cutting or cultured for either 48 or 96 h.

**Slice number**	**Culture time (hours)**	**RNA (ng/μl)**	**Impurities (A260)**	**Residue (%)**	**A260**	**A260/A280**
1	0	11.2	0.43	1.1	0.71	1.83
3	0	83.2	0.00	0.4	2.08	2.06
5	0	121.2	0.01	0.3	4.28	2.08
1	48	N/A	0.11	21.7	0	0
3	48	51.4	0.02	0.9	1.32	2.13
5	48	91.0	0.00	0.5	2.27	2.10
1	96	N/A	0.00	2.6	0.00	0
3	96	11.3	0.01	0.8	0.81	2.17
5	96	32.3	0.02	0.7	0.90	2.11

### Spleen Slices Cultured for 48 h *in vitro* Are Responsive to Exogenous Stimuli

**Figure 7:**
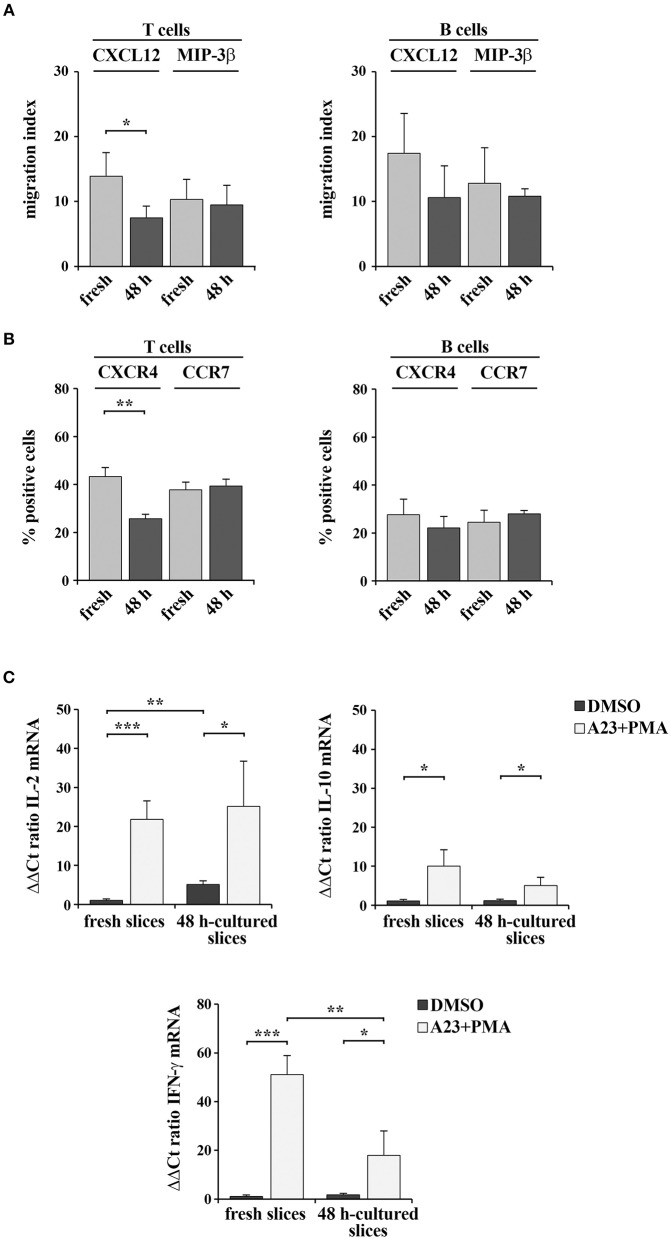
48 h-culture does not significantly alter spleen slice responsiveness to exogenous stimuli. **(A)** Migration of T and B cells from intact spleen slices, either freshly prepared or cultured *in vitro* for 48 h and placed on the upper wells of Boyden chambers. Slice-resident T and B cells were allowed to migrate for 3 h toward the lower wells containing 100 ng/ml CXCL12 or MIP3-β. Cells recovered from the lower wells were stained with PE anti-CD3 and FITC anti-CD19 antibodies and counted by flow cytometry. The data, obtained on duplicate samples from each spleen slice, are presented as mean migration index (ratio migrated cells in chemokine-treated vs. untreated samples) ± SD. **(B)** Flow cytometric analysis of the percentages of CXCR4^+^ or CCR7^+^ T lymphocytes (stained with anti-CD3 antibodies) and CXCR4^+^ or CCR7^+^ B lymphocytes (stained with anti-CD22 antibodies) in spleen slices disgregated either immediately after cutting (fresh) or after *in vitro* culturing for 48 h. The data are presented as the percentage of either CXCR4^+^ or CCR7^+^ cells over total CD3^+^ or CD22^+^ cells (*n* = 3). **(C)** Quantitative RT-PCR analysis of IL-2, IL-10, and IFN-γ mRNA in samples from 3 spleen slices stimulated for 6 h with 500 ng/ml A23187 (A23) and 100 ng/ml PMA (A23+PMA) or with carrier (DMSO) either immediately after cutting or after *in vitro* culturing for 48 h and then homogenized for RNA extraction. The relative gene transcript abundance was determined on triplicate samples using the ddCt method. *Gapdh* was used as housekeeping gene. Mean ± SD. Student's *t* test; ***p ≤ 0.001; **p ≤ 0.01; *p ≤ 0.05.

We assessed whether 48-h *in vitro* culturing preserves the responsiveness of spleen slices to exogenous stimulation. The chemotactic ability of cells within spleen slices either immediately after cutting or cultured for 48 h at 37°C was analyzed by Transwell assays. Intact slices were placed on the upper wells of Boyden chambers and cells were allowed to migrate to the lower wells toward the chemokines CXCL12 or MIP-3β, which bind the respective lymphocyte-specific receptors CXCR4 and CCR7 (17). Migrated T and B cells were then stained with anti-mouse CD3 PE and anti-mouse CD19 FITC antibodies and quantified by flow cytometry. As shown in Figure 7A, chemotaxis of both T and B cells toward MIP-3β (Figure 7A) was barely affected by *in vitro* culturing, as was surface CCR7 (Figure 7B). CXCL12-dependent chemotaxis, although still clearly detectable in 48 h-cultured slices, was slightly decreased (Figure 7A), however this was likely the consequence of a loss of surface CXCR4 rather than a loss of responsiveness (Figure 7B). Hence, cells residing in slices cultured *in vitro* for 48 h maintain their responsiveness to chemotactic stimuli.

Freshly cut and 48 h-cultured spleen slices were also analyzed for their ability to respond to the non-specific mitogenic combination of the Ca^2+^ ionophore A23187 and PMA. As a read-out of cell stimulation, we quantified by qRT-PCR the mRNA levels of the cytokines IL-2, IL-10, and IFN-γ, which are expressed in lymphocytes and stromal cells following mitogenic stimulation (18, 19). As shown in Figure 7C, both freshly cut slices and slices cultured *in vitro* for 48 expressed enhanced amounts of the analyzed cytokines following stimulation. Interestingly, while IFN-γ expression was lower in the 48-h *in vitro* cultured slices compared to the freshly cut ones, the expression of both IL-2 and IL-10 was unaffected by *in vitro* culturing (Figure 7C). Collectively, these results demonstrate that spleen slices cultured *in vitro* for 48 h are suitable for functional assays and retain the sensitivity to exogenous stimuli similar to freshly prepared spleen slices.

## Discussion

Here we set up a method that allows for the efficient precision-cut of mouse spleens to obtain non-damaged, live slices for organotypic spleen culture. We furthermore optimized the *in vitro* culture of these slices. We show that spleen slices can be maintained in culture for 48 h without marked loss of organ architecture and with a minimal loss of cell viability, which allows to perform functional assays on slices treated *in vitro*. Moreover, the quality of the RNA isolated from cell homogenates of 48 h-cultured slices is sufficient to allow for the amplification of low-abundance chemokine transcripts.

Organotypic culture technology is routinely used to perform organotypic culture of central nervous system sections (20, 21). However, its application to lymphoid organs has lagged behind. We found only one paper describing this technique on human spleen and lymph node biopsies, where the authors obtain slices not <400 μm-thick which survive in culture for up to 1 week (4). The method we propose, which produces 230 μm-thick spleen slices or lower, represents a new application of this technique that meets the need to provide an easy-to-handle way to simulate the 3-D context of the immune system where all cellular components are represented in the respective physiological locations. The limited thickness of the slices that we obtain will be useful for treatments *in vitro*, which can easily reach the whole depth of the slices. However, the standard conditions that we apply for the *in vitro* culture of spleen slices do not allow us to maintain tissue slices viable for longer than 48 h, as instead reported by Hoffmann and colleagues for human lymphoid biopsies (4), possibly due to insufficient oxygen perfusion. Organotypic culture often requires dedicated culture media supplemented with specific nutrients or growth factors (22, 23). Optimizing the *in vitro* culture protocol may help prolonging the viability of vibratome-generated, thin spleen slices for longer-term functional assays *in vitro*.

Studying the response of immune cells within their microenvironment has become a central requirement for the development of personalized immunotherapy-based treatments against cancer (24). It is noteworthy that, among recent technical advances, new miniaturized “organ-on-a-chip” cultures were introduced in an attempt to recapitulate *in vitro* the organ environment by mimicking blood flow through microfluidic systems (25, 26). However, the high costs of this technique preclude its generalized exploitation. By comparison, the organotypic culture represents a cheap, easy-to-handle method to study *in vitro* the interplay among the cellular components of the stromal microenvironment in both physiological and pathological settings and to test the immune cell response to drugs.

## Data Availability Statement

All datasets generated for this study are included in the article/Supplementary Material.

## Ethics Statement

The animal study was reviewed and approved by the Italian Ministry of Health (197/2015-PR) and OPBA, University of Siena.

## Author Contributions

LP, FF, NM, NC, VT, and FL performed the work. LP and CB wrote the manuscript. LB, GP, and IC contributed to set up the spleen slice culture conditions.

### Conflict of Interest

The authors declare that the research was conducted in the absence of any commercial or financial relationships that could be construed as a potential conflict of interest.
