# Supplementary material for: Optimization of Organotypic Cultures of Mouse Spleen for Staining and Functional Assays
Source: Front Immunol. 2020 Mar 24;11:471. doi: 10.3389/fimmu.2020.00471 (PMC7105700; doi:10.3389/fimmu.2020.00471)
Supplement: Supplementary file 1 [file Data_Sheet_1.PDF]

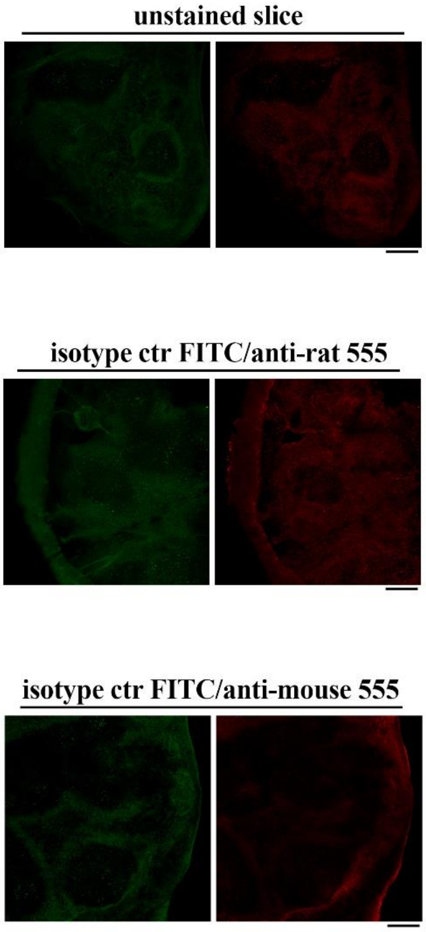

Legend to Supplementary Figure 1. Immunofluorescence analysis of spleen slices immediately after cutting, permeabilized and either left unstained or labeled with anti-Rat 555 (red) / isotype control FITC (green) or with anti-Mouse 555 (red) / isotype control FITC (green) antibodies. Median optical sections are shown. Size bar, 200 µm.
